# Dll1 Can Function as a Ligand of Notch1 and Notch2 in the Thymic Epithelium

**DOI:** 10.3389/fimmu.2022.852427

**Published:** 2022-03-17

**Authors:** Ken-ichi Hirano, Hiroyuki Hosokawa, Takashi Yahata, Kiyoshi Ando, Masayuki Tanaka, Jin Imai, Masaki Yazawa, Masato Ohtsuka, Naoko Negishi, Sonoko Habu, Takehito Sato, Katsuto Hozumi

**Affiliations:** ^1^ Department of Immunology, Tokai University School of Medicine, Isehara, Japan; ^2^ Institute of Medical Sciences, Tokai University, Isehara, Japan; ^3^ Department of Innovative Medical Science, Tokai University School of Medicine, Isehara, Japan; ^4^ Department of Hematology and Oncology, Tokai University School of Medicine, Isehara, Japan; ^5^ Support Center of Medical Research and Education, Tokai University School of Medicine, Isehara, Japan; ^6^ Divison of Gastroenterology and Hepatology, Tokai University School of Medicine, Isehara, Japan; ^7^ Department of Molecular Life Science, Tokai University School of Medicine, Isehara, Japan; ^8^ Department of Immunology, Juntendo University School of Medicine, Tokyo, Japan

**Keywords:** delta-like 1, delta-like 4, Notch1, Notch2, thymus, phylogenesis

## Abstract

T-cell development in the thymus is dependent on Notch signaling induced by the interaction of Notch1, present on immigrant cells, with a Notch ligand, delta-like (Dll) 4, on the thymic epithelial cells. Phylogenetic analysis characterizing the properties of the Dll4 molecule suggests that Dll4 emerged from the common ancestor of lobe- and ray-finned fishes and diverged into bony fishes and terrestrial organisms, including mammals. The thymus evolved in cartilaginous fishes before Dll4, suggesting that T-cell development in cartilaginous fishes is dependent on Dll1 instead of Dll4. In this study, we compared the function of both Dll molecules in the thymic epithelium using *Foxn1-cre* and *Dll4*-floxed mice with conditional transgenic alleles in which the *Dll1* or *Dll4* gene is transcribed after the cre-mediated excision of the stop codon. The expression of Dll1 in the thymic epithelium completely restored the defect in the *Dll4*-deficient condition, suggesting that Dll1 can trigger Notch signaling that is indispensable for T-cell development in the thymus. Moreover, using bone marrow chimeras with *Notch1*- or *Notch2*-deficient hematopoietic cells, we showed that Dll1 is able to activate Notch signaling, which is sufficient to induce T-cell development, with both the receptors, in contrast to Dll4, which works only with Notch1, in the thymic environment. These results strongly support the hypothesis that Dll1 regulates T-cell development *via* Notch1 and/or Notch2 in the thymus of cartilaginous fishes and that Dll4 has replaced Dll1 in inducing thymic Notch signaling *via* Notch1 during evolution.

## Introduction

The Notch system—highly conserved from invertebrates to mammals—regulates lineage specification during organogenesis in various cell types (1, 2). These signals travel between adjacent cells *via* the specific interaction of the Notch receptors with their ligands, belonging to the delta-like and jagged protein families. Their specific binding results in the proteolysis of Notch and the movement of the Notch intracellular domain (NICD) into the nucleus, where the active fragment of Notch functions as a scaffold protein with the DNA-binding protein, RBPJ, and transcriptional activators. It is an essential component of signal transduction.

During the differentiation of hematopoietic cells, only the T-cell lineage requires a specialized environment in the thymus, where the immigrant cells receive Notch signaling induced by the interaction of Notch1 on the immigrant cells and the Notch ligand Delta-like 4 (Dll4) on the thymic epithelial cells (3–5). Evidently, within the four essential factors, namely, Ccl25, Cxcl12, Scf, and Dll4, Dll4 provides the key stimulus that determines the fate of T cells in a *Foxn1*-deficient background (6). Moreover, as the expression of Dll4 is maintained by the three-dimensional structure of thymic epithelial cells, their monolayer cultures lose Dll4 expression and the ability to support T-cell development *in vitro* (7, 8). Thus, Dll4 defines the thymus as the site of T-cell development.

The expression of Dll4 in thymic epithelial cells is induced by Foxn1, a transcription factor essential for thymic development (9–11), *via* its interaction with the enhancer region of the *Dll4* locus (12) that is shared with Foxn4 in endothelial cells (13). The phylogenetic significance of these transcription factors in thymic development has been analyzed in detail (9–11). Interestingly, Foxn1 is expressed alone in the mammalian thymic epithelium, while it is co-expressed with Foxn4 in the thymus of cartilaginous fishes, inducing a characteristic structure that supports B-lymphopoiesis. Therefore, the thymic environment appears to have changed during evolution (10, 11). Moreover, sex hormones regulate the expression of Dll4. Steroid administration causes thymocyte death and thymic atrophy, and conversely, sex steroid ablation increases thymopoiesis. This could be explained by the fact that sex hormones characteristically suppress the expression of Dll4 in thymic epithelial cells and that sex steroid ablation increases the expression of Dll4, resulting in efficient T-cell development in the thymus (14). Thus, the expression of Dll4 in thymic epithelial cells may be a clinical target to improve the T-cell supply from aged thymuses.

It is important to note that the *Dll4* gene is absent in the early jawed vertebrates (cartilaginous fishes), where only the *Dll1* gene is present (9). This is consistent with the presence of the *Dll1* ortholog gene transcript in the epithelium of the thymus-like structure in the gills of lamprey larvae (15). Therefore, when the thymus first appeared in early jawed vertebrates as a site of T-cell development, Dll1, and not Dll4, may have predominated the thymic environment. In mice, Notch1 is an essential partner of Dll4 for T-cell development in the thymus, but Notch2 is also detected in hematopoietic progenitors immediately after their thymic migration (16). Therefore, it is unclear why thymic immigrants lacking Notch1 cannot receive Dll-mediated Notch signaling *via* Notch2 (17). In some cases, a specific combination of Notch and Notch ligands may function selectively in a context-dependent manner (18). However, whether Notch2 can mediate Notch signaling and contribute to T-cell development in the thymus has not been examined.

We have previously revealed the physiological significance of Dll4 in murine T-cell development in the thymus (4, 16). Dll1 is scarcely detected in the thymic environment (19, 20) and dispensable for triggering T-cell development in the thymus (19). Moreover, the superiority of Dll4 over Dll1 for T-cell induction has been shown (21). We attributed the functional characteristics of Dll4 to the mobility of the loop structure within the module at the N-terminus of Notch ligand (MNNL) domain at the tip of the ligand and showed that the DOS motif observed in the first/second epidermal growth factor (EGF)-like repeat present in Notch ligands—except Dll4—augments the activity of Dll4 using their chimeric molecules (21). Therefore, Dll family members bind to Notch and trigger signaling differently based on their structural features. In this study, we showed the phylogenetic interrelationships of the *Dll1* and *Dll4* homologous genes and discussed the emergence and evolution of both the genes based on the properties of the MNNL and first/second EGF-like repeat regions that characterize the Dll molecules. Furthermore, we demonstrated that Dll1, which likely functions as a Notch ligand during thymus emergence, can support T-cell development in thymic epithelial cells with both Notch1 and Notch2, whereas Dll4 only works with Notch1, in our experimental model.

## Results

### The *Dll4* Gene Identified in Coelacanth Shares Distinctive Characteristics With the *Dll1* and *Dll4* Genes in Terrestrial Organisms

**Figure 1:**
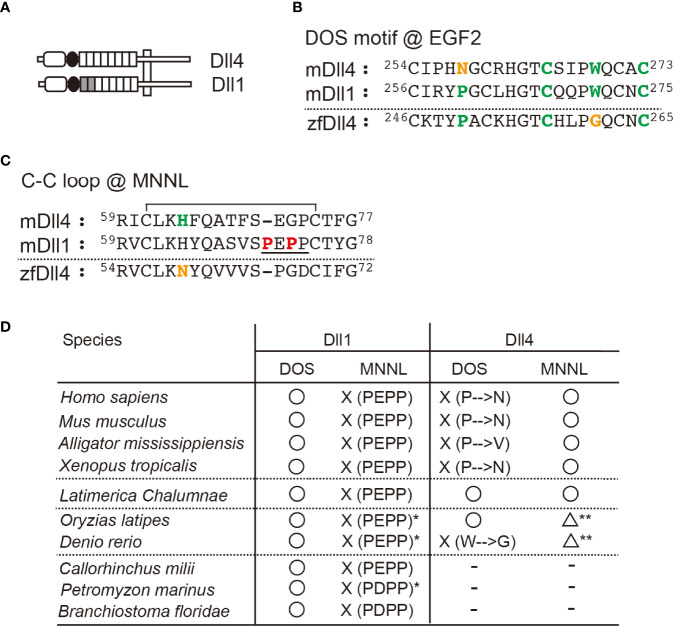
Characteristic features of Dll1 and Dll4. **(A)** Schematic structure of Dll1 and Dll4. The MNNL and DSL domains are represented by an open rectangle with round corners and a filled circle, respectively. The EGF-like repeat is shown by square, and the first and second repeats retaining the DOS motif in Dll1 are filled. Both Notch ligands are present on the cell membrane (vertical square). **(B, C)** Amino acid (AA) sequence comparison of the DOS motif in the second EGF-like repeat **(B)** and the C–C loop in MNNL domain **(C)** between murine (m) Dll4, Dll1, and zebrafish (zf) Dll4. Numbers on the AA sequences represent the position from the N-terminus. The AAs in the DOS motif (**B**, bold green) and their substitution (**B**, bold orange) are labeled. Similarly, histidine in the C–C loop **(C)**, contributing to the direct binding with Notch (**C**, bold green) and its substitution (**C**, bold orange), is also labeled. The C–C loop in mDll1 contains a characteristic proline-rich AA region (**C**, underlined with unique prolines, bold red). Line over the sequence represents the disulfide bridge between cysteine residues (61st to 74th in mDll4). **(D)** Characterization of the conservation of DOS motif (DOS) and the predicted functionality of MNNL domain (MNNL) in Dll1 and Dll4 homologs in various species. ○, functional; ☓, non-functional; △, attenuated (predicted); −, absent. *One of the orthologs retains PEPP or PDPP residues. **The histidine residue (bold green in **C**) at the C−C loop pf MNNL domain is replaced with asparagine (bold orange in **C**).

*Dll1* and *Dll4* are conserved in bony fish and terrestrial organisms, including mammals. These Notch ligands share structural characteristics, but mammalian Dll4s do not retain the DOS motif necessary for the binding of Dll1 to Notch1 due to the substitution of Pro in the motif to Asn at the second EGF-like repeats (**Figures 1A, B** **)** (2, 16). On the other hand, the N-terminal MNNL region of murine Dll4 that contains a loop structure with a wide range of motion directly contributes to binding with Notch1 (22). In contrast, that of murine Dll1 loses the ability to move widely because of its rigidity due to the sequential presence of unique prolines (**Figure 1C**) (21). Therefore, Dll molecules seem to bind to Notch1 in different regions. To examine the characteristics of Dll1 and Dll4 molecules during evolution, we sorted the genes of Dll family members from the National Center for Biotechnology Information (NCBI) database according to their homologies and formed a phylogenetic tree (**Supplementary Figure 1**). This analysis indicated that the *Dll4* gene first emerged in bony fishes, while the *Dll1* genes were identified from amphioxus (*Branchiostoma floridae*) and vertebrates, which is consistent with a previous report (9). In the *Dll4* genes, the DOS motif maintained in many bony fishes (23 species, including medaka fish, **Figure 1D**) is different from the one in terrestrial organisms, with some exceptions (zebrafish, arowana, and Japanese pufferfish) that have another substitution in the DOS motif (Trp to Gly, **Figure 1D**). However, one of the critical residues at the interface of the MNNL region (22), His, was substituted with Asn in all bony fishes (**Figure 1D**), suggesting that the binding ability of MNNL of Dll4 in bony fishes to Notch is likely to be reduced. In contrast, the *Dll1* genes are highly conserved with the DOS motif and unique Pro residues in the C–C loop of the MNNL region (**Figure 1D**).

The *Dll4* gene found in coelacanth—the famous lobe-finned fish—surprisingly encodes both the DOS motif and His in the MNNL region, which is different from those in terrestrial organisms and bony fishes (**Figure 1D**). We previously showed that a murine Dll4-derived chimera with Dll1-derived first and second EGF-like repeats containing the DOS motif exhibited stronger activity to trigger Notch signaling than the original Dll4 (21). Therefore, Dll4 in coelacanths should induce a stronger Notch signal than in other species. It is understood that ray- and lobe-finned fish that evolved into tetrapods share a common ancestor, and coelacanths have shown a slow rate of molecular and morphological evolution (23). As Dll4 in coelacanths is predicted to show intermediate characteristics between those of tetrapods and bony fishes, it was estimated that the *Dll4* gene first appeared in a form similar to the coelacanth one in the common ancestor of lobe- and ray-finned fishes and changed to its respective forms in terrestrial organisms and bony fishes during evolution.

The thymus, a primary lymphoid organ essential for T-cell development, emerged in jawed vertebrates approximately 500 million years ago (9). As the *Dll4* gene has never been identified in cartilaginous fish (*Callorhinchus milii*, **Figure 1D**), Dll1 is likely to function as a Notch ligand on the thymic epithelium in gnathostomes ancestors. However, it is unclear whether Dll1 can function as a Notch ligand on the thymic epithelium and support T-cell development in the thymus, and which Notch receptor actually interacts with Dll1 in thymic immigrants.

### Dll1 Can Support the T-Cell Development in the Murine Thymus

**Figure 2:**
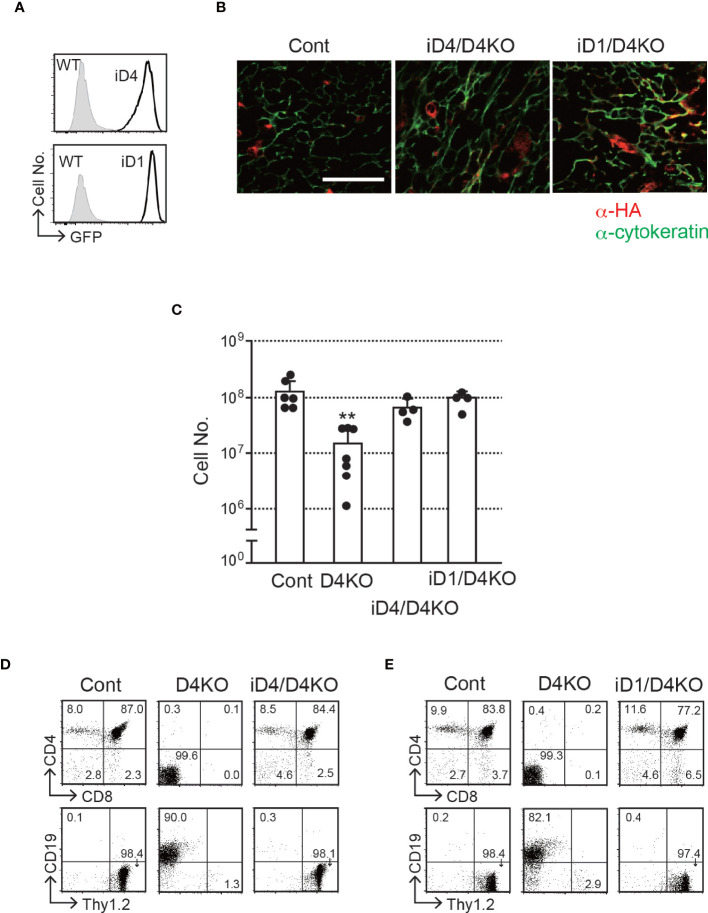
T-cell development in the thymus with epithelial cells expressing exogenous Dll4 or Dll1 in *Dll4*-deficient background. **(A)** GFP expression, transcripts driven by CAG promoter at the *Rosa26* locus of iD1 and iD4 mice, was detected in EpCAM^+^PDGFRα^-^ epithelial cells obtained from fetal (E15.5) thymus using flow cytometry. Open histograms indicate GFP expression of *Rosa26^floxedGFP-Dll4^* (iD4) or *Rosa26^floxedGFP-Dll1^* (iD1) mice, and filled histograms indicate the intrinsic fluorescence of the identical cell population of control (WT) mice. **(B)** Representative results of immunofluorescence microscopy analysis of the thymus from *Dll4^f/f^* (Cont), iD4*Dll4^f/f^* FoxN1-Cre (iD4/D4KO), or iD1*Dll4^f/f^* FoxN1-Cre (iD1/D4KO) mice stained with anti-HA (red) and anti-cytokeratin (green) antibodies are shown. Intense and widespread red staining (anti-HA Ab) were nonspecific staining. Scale bar: 50 μm. **(C)** Thymic cellularity (mean ± SD) of 8- to 12-week-old *Dll4^f/f^* (Cont, n=6), *Dll4^f/f^* FoxN1-Cre (D4KO, n=7), iD4*Dll4^f/f^* FoxN1-Cre (iD4/D4KO, n=4), or iD1*Dll4^f/f^* FoxN1-Cre (iD1/D4KO, n=4) mice. **p<0.01 by Student’s *t*-test. **(D, E)** Flow cytometric analysis of thymocytes from the mice shown in panel **(B)** was performed. Thymocytes were stained with mAbs against surface molecules as indicated. Numbers in the profiles indicate the relative percentages for each quadrant. Results represent more than three independent experiments **(A, B, D, E)**.

To explore the ability of Dll1 to trigger Notch signaling in the thymic epithelium, we used conditional transgenic (Tg) mice, in which one copy of the *Dll1* or *Dll4* gene was transcribed by the CAG promoter after Cre-dependent gene deletion of floxed *GFP* cDNA at the *Rosa26* locus (hereafter referred to as iD1 and iD4 Tg) (21). In these Tg mice, we were able to detect the expression of GFP in EpCAM^+^ PDGFRα^−^ thymic epithelial cells derived from the fetal murine thymus (**Figure 2A**), indicating that the CAG promoter substantially transcribed the inserted gene cassette containing *Dll1* or *Dll4* cDNA in the *Rosa26* allele. A small difference in fluorescence intensity could be due to the difference between *Dll1* and *Dll4* cDNA sequences because one copy of either of the cDNAs was inserted into the same site of the *Rosa26* locus. After breeding *Foxn1-Cre* and *Dll4-floxed* mice, consistent with GFP expression, exogenously expressed HA-tagged Dll4 or Dll1 was detected in cytokeratin^+^ thymic epithelium (**Figure 2B**). Thus, we examined the effect of exogenous expression of Dll4 or Dll1 on T-cell development under endogenous *Dll4*-deficient thymic conditions in which T-cell development has been completely impaired (4, 5). The reduction in total cell numbers observed in the *Dll4*-deficient thymic lobe was completely reversed by the expression of exogenous Dll4 or Dll1 (**Figure 2C**). Consistent with the cell numbers, efficient T-cell development was detected with exogenous Dll4 or Dll1 without endogenous Dll4 in the thymic epithelium (**Figures 2D** **, E** and **Supplementary Figure 2**). Thus, Dll1 can function as a Notch ligand to support T-cell development in the thymus. However, it remains unclear which Notch receptor binds to exogenous Dll1 and mediates signal transduction in T-cell progenitors in these Tg mice.

### Both Notch1 and Notch2 Are Detected on the Common Lymphoid Progenitors in the Murine Bone Marrow

**Figure 3:**
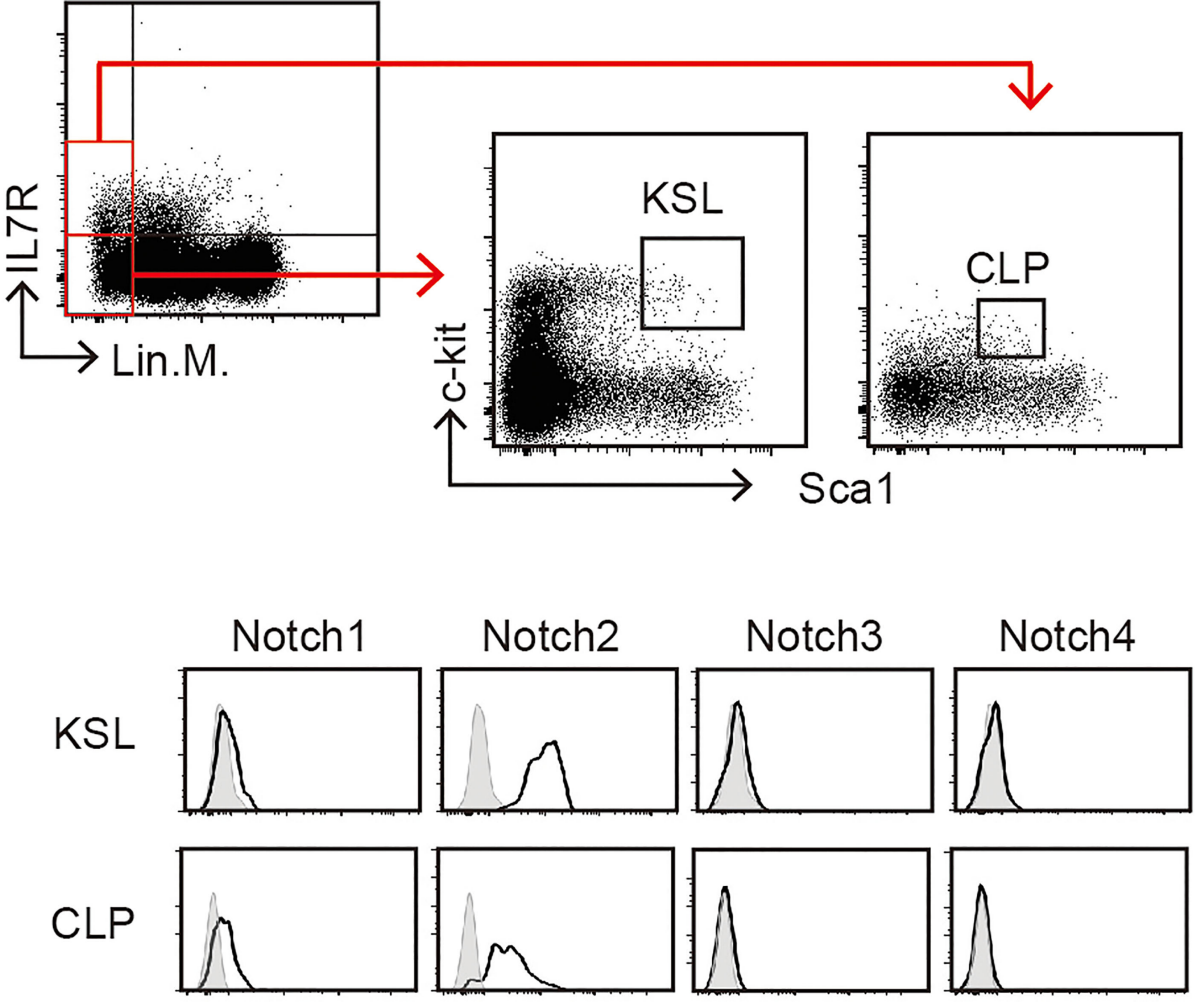
Expression of Notch receptors on immature hematopoietic cells in the bone marrow. Bone marrow (BM) cells of C57BL/6 mice were subdivided into KSL (lineage markers^-^, c-kit^+^, and Sca1^+^) and CLP (lineage markers^-^, c-kit^low^, and Sca1^low^) populations (upper panels) and analyzed for Notch expression using flow cytometry (lower panels). Open histograms indicate staining with mAbs recognizing Notch1, Notch2, Notch3, or Notch4. Filled histograms indicate staining with control hamster IgG. These profiles represent at least three independent experiments.

In mammals, of the four Notch receptors identified, three—Notch1, Notch2, and Notch3—have been detected in the blood cells (24). The phenotypes of conditional KO mice demonstrated that Notch1 and Notch2 mainly contribute to the development of hematopoietic cells (3, 25). Here, we confirmed the expression of Notch receptors on pre-thymic T-cell progenitors in the murine bone marrow (**Figure 3**). As a population that includes hematopoietic stem cells, weak but detectable expression of Notch1 and high expression of Notch2 were detected in lineage marker-negative c-kit^+^Sca1^+^ (KSL) cells. During differentiation toward the lymphoid lineage, the Notch1 expression increased, but that of Notch2 decreased, and both receptors were clearly detected in the common lymphoid progenitors (lineage marker-negative c-kit^low^Sca1^low^IL-7R^+^). These profiles were consistent with those obtained in a similar population of the murine fetal liver (26). We did not observe Notch3 and Notch4 expression in these progenitors. In addition, both Notch1 and Notch2 are co-expressed in the earliest stage of T-cell progenitors in the thymus (early T-cell progenitors) (16, 27, 28). These results suggest that thymic immigrants express both Notch1 and Notch2 on their surfaces and receive Notch signaling *via* both receptors. We have reported that signal transduction from the active intracellular fragment of Notch1 or Notch2 is sufficient for the initiation of T-cell lineage development (29) and that Notch2 complements Notch1 to mediate inductive signaling for T-cell development in pro-T stages (27). Therefore, we expected that both Notch1 and Notch2 could contribute to the T-cell development in the thymus, especially with exogenous Dll1 that preferentially stimulates Notch2-mediated signal transduction in other cell types (18, 19).

### Both Dll1 and Dll4 Interact With Notch1 in the Thymus

**Figure 4:**
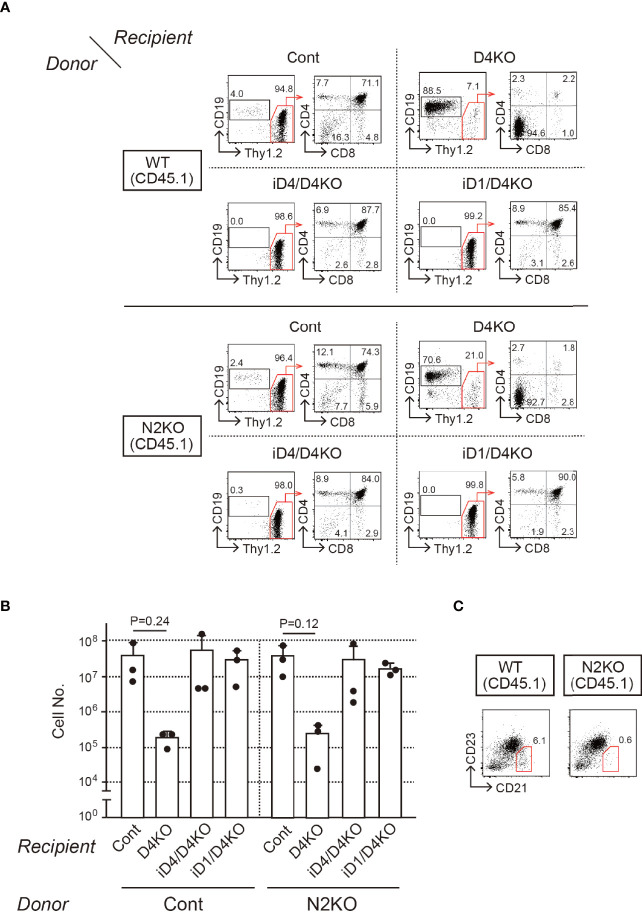
T-cell development from *Notch2*-deficient BM cells in the thymus of iD4 and iD1 mice. Age-matched control (WT) or *Notch2*-deficient (N2KO) BM cells were obtained from *Notch2^f/f^* or *Rosa26^CreERT2^Notch2^f/f^* mice (CD45.1) 1 week after the administration of tamoxifen. BM chimeric mice were prepared in irradiated (6 Gy) C57 BL/6 mice (CD45.2) with *Notch2*-deficient BM and analyzed 4 weeks after the reconstitution (**Supplementary Figure 3A**). **(A)** Flow cytometric analysis was performed using the thymocytes from BM chimeric mice with control (WT) or *Notch2*-deficient (N2KO) BM cells (*Donor*) in *Dll4^f/f^* (Cont), *Dll4^f/f^* FoxN1-Cre (D4KO), iD4*Dll4^f/f^* FoxN1-Cre (iD4/D4KO), or iD1*Dll4^f/f^* FoxN1-Cre (iD1/D4KO) mice as the recipients (*Recipient*). Numbers in the profiles indicate the relative percentages, in CD45.1^+^ cells (left panels, CD19 vs. Thy1.2) and CD45.1^+^Thy1.2^+^ cells (right panels, CD4 vs. CD8), for each quadrant or fractions. **(B)** Thymic cellularity (mean ± SD) of BM chimeric mice in *Dll4^f/f^* (Cont, n=3), *Dll4^f/f^* FoxN1-Cre (D4KO, n=3), iD4*Dll4^f/f^* FoxN1-Cre (iD4/D4KO, n=3), or iD1*Dll4^f/f^* FoxN1-Cre (iD1/D4KO, n=3) mice is shown. There are no statistically significant differences found between control and *Notch2*-deficient BM cells by Student’s *t*-test. Each closed circle indicates the number of thymocytes (CD45.1) in each mouse. **(C)** Representative CD21/CD23 profiles in the donor-derived B cells (CD45.1^+^B220^+^) obtained from the spleen of BM chimeric mice with control (WT) or *Notch2*-deficient (N2KO) BM cells in *Dll4^f/f^* mice are shown. The red polygons and numbers in the profiles indicate the MZB cell fraction and their frequencies. Results represent three independent biological replicates **(A, C)**.

To reveal functional Notch receptors for exogenous Dll4 and Dll1 on T-cell progenitors, we prepared BM chimeras in iD1/iD4 Tg mice with *Notch1*- or *Notch2*-deficient bone marrow cells and examined their T-cell development in the thymus. *Notch2*-deficient BM cells were prepared from *Notch2* ^f/f^ mice (23) with *Cre/ERT2* knock-in allele in the *Rosa26* locus (30) after tamoxifen administration and then transferred into irradiated iD1 or iD4 Tg mice with a *Dll4*-deficient background (**Supplementary Figure 3A**). Six weeks later, T-cell development in the thymus with *Notch2*-deficient T-cell progenitors was completely rescued by exogenous Dll4 and Dll1 and was comparable to that in WT mice (**Figures 4A, B** **)**. In BM chimeras with *Notch2*-deficient BM cells, CD21^high^CD23^-^ splenic marginal zone B (MZB) cells disappeared selectively (**Figure 4C**), which is consistent with previous reports using mice with B-cell-specific deletion of *Notch2* (25) or systemic disruption of *Dll1* (19). These results suggest that both Dll1 and Dll4 can trigger Notch signaling *via* Notch1 in the thymus.

### Only Dll1 Can Induce T-Cell Development With Notch2 in the Thymus

**Figure 5:**
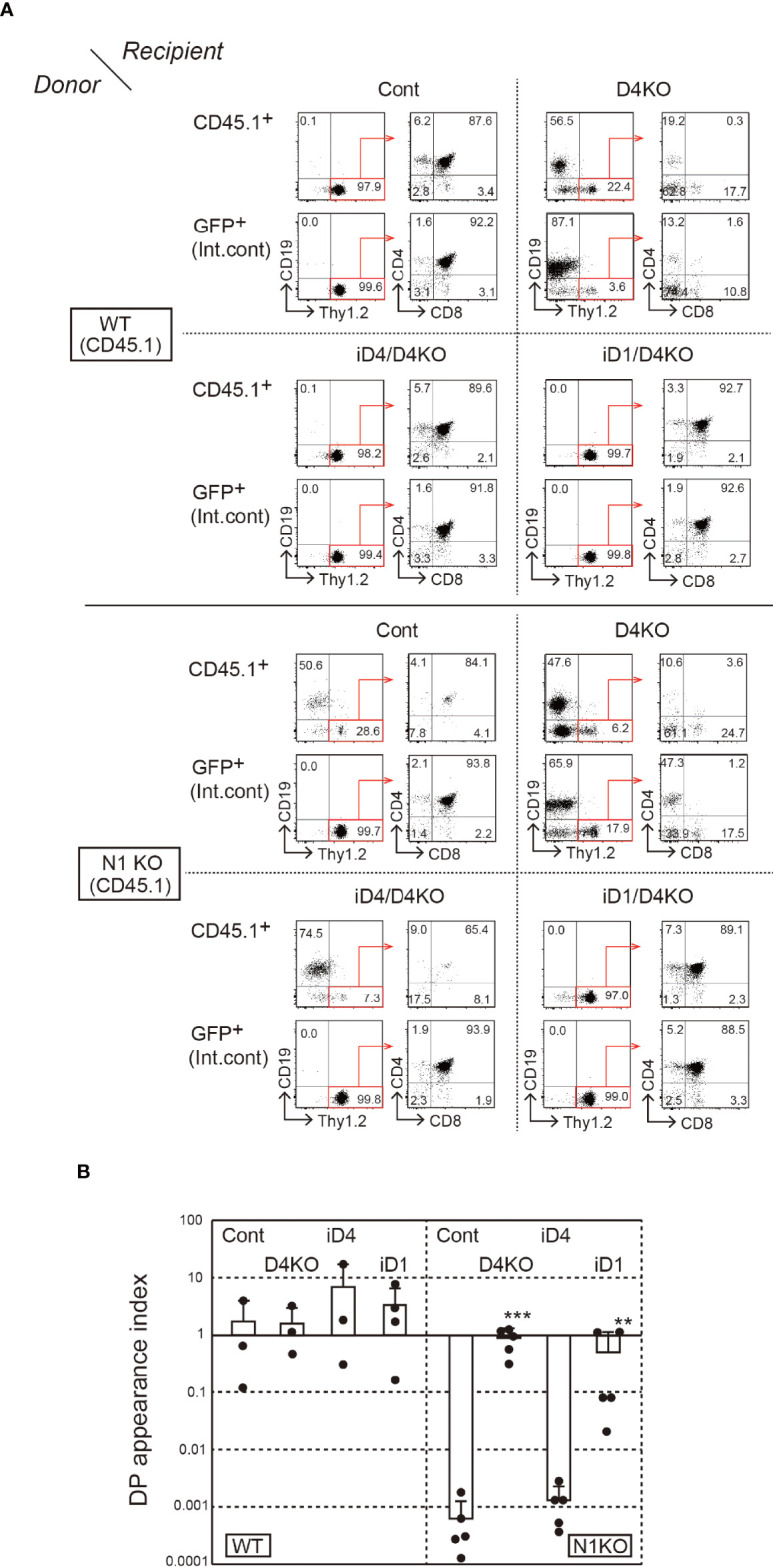
T-cell development from *Notch1*-deficient BM cells in the thymus of iD4 and iD1 mice. The primary BM chimeras were prepared in irradiated WT (CD45.2) mice with BM cells from *Notch1^f/f^* (WT, CD45.1) or *Rosa26^CreERT2^Notch1^f/f^* (N1KO, CD45.1) mice and GFP Tg mice. The control and *Notch1*-deficient BM cells were obtained from the primary BM chimeras after the administration of tamoxifen and serially transferred into *Dll4^f/f^* (Cont), *Dll4^f/f^* FoxN1-Cre (D4KO), iD4*Dll4^f/f^* FoxN1-Cre (iD4/D4KO), or iD1*Dll4^f/f^* FoxN1-Cre (iD1/D4KO) mice. Four weeks after the reconstitution, thymocytes were analyzed (**Supplementary Figure 3B**). **(A)** Flow cytometric analysis was performed using the thymocytes from the secondary BM chimeric mice. Numbers in the profiles indicate the relative percentages, in CD45.1^+^ or GFP^+^ cells (internal control, Int. cont) (left panels, CD19 vs. Thy1.2) and CD45.1^+^Thy1.2^+^ or GFP^+^Thy1.2^+^ cells (right panels, CD4 vs. CD8), for each quadrant or fractions. Results represent at least three independent biological replicates. **(B)** The efficiencies of the appearance of CD4^+^CD8^+^ (DP) thymocytes derived from control (WT) or *Notch1*-deficient (N1KO) BM cells were examined. DP appearance index was calculated as the ratio of CD45.1^+^/GFP^+^ DP thymocytes and CD45.1^+^/GFP^+^ B220^+^ B cells in lymph node (mean ± SD; WT as donor; Cont, n=3; D4KO, n=3; iD4/D4KO, n=3; iD1/D4KO, n=4; N1KO as donor; Cont, n=5; D4KO, n=5; iD4/D4KO, n=5; iD1/D4KO, n=5). The data were collected from three independent experiments. Each closed circle indicates the index in each mouse. **p < 0.01, ***p < 0.001 by Mann–Whitney *U*-test.

Next, we prepared BM chimeras with *Notch1*-deficient BM cells. However, systemic depletion of *Notch1* affects the survival of mice, and it is difficult to obtain *Notch1*-deficient BM cells. Thus, we performed sequential transplantation of the BM (**Supplementary Figure 3B**). First, irradiated WT host mice (CD45.2^+^) were reconstituted with BM cells derived from *Notch1* ^f/f^ mice (31) with *Cre/ERT2* knock-in allele in *Rosa26* locus; then, they were treated with tamoxifen. After a week of the last tamoxifen treatment, *Notch1*-deficient BM cells were secondarily transferred into iD1/iD4 mice with a *Dll4*-deficient background. In that case, it was difficult to control the efficiency of thymopoiesis reconstitution. Therefore, we used GFP^+^ BM cells as an internal control at the first transplantation and evaluated T-cell development relative to the GFP^+^ control in secondary BM chimeras. In these experiments, the majority of *Notch1*-deficient BM cells differentiated into CD19^+^ B-lineage cells in the thymus under control and *Dll4*-deficient conditions (**Figure 5A**). Moreover, similar developmental patterns were observed in iD4 mice, indicating that Dll4 does not support Notch2-mediated T-cell development in the thymus (**Figure 5A**). In contrast, *Notch1*-deficient BM cells were able to differentiate into T-lineage cells, including CD4/CD8 double-positive (DP) and single-positive (SP) cells, in the thymus of iD1 mice, but not into B-lineage cells—the default phenotype in the absence of Notch signaling (**Figure 5A**). These phenotypes were also confirmed in the inguinal lymph nodes (**Supplementary Figure 4**). However, T-cell development of *Notch1*-deficient cells supported by Dll1 may be less efficient than that of WT cells because, in some cases, the ratios of the number of the DP cells derived from *Notch1*-deficient cells were lower than those from the GFP^+^ internal control (**Figure 5B**). We observed spontaneous differentiation of Thy1.2^+^ T-lineage cells in the *Dll4*-deficient thymus. This phenotype would be caused by the inefficient differentiation of B cells in the sequential BM transplantation experiments. In some cases, GFP^+^ cells did not efficiently differentiate into SP cells, which might be due to the excess expression of GFP. These results suggest that Dll1 on the epithelial cells, but not Dll4, interacts with Notch2 on the immigrant cells in the thymus and retains its superiority over Dll4 for induction of T-cell development *via* Notch2.

### Pairing of Dll1/Notch2 Is Present in the Thymus of Elephant Sharks

After estimating the time of emergence of the two Dll molecules, we formed a phylogenetic tree of *Notch1* and *Notch2* genes to determine when the Notch receptors evolved to comprise multiple molecules (**Supplementary Figure 5**). Like with the emergence of the *Dll4* gene in coelacanths and bony fishes, the *Notch2* gene was first recognized in cartilaginous fishes and has been passed on to coelacanths and bony fishes. On the other hand, the *Notch1* genes, like the *Dll1* genes, were identified in all vertebrates, including lampreys and amphioxus. Notably, the *Notch1* gene in the elephant shark (*Callorhinchus milii*) contains shorter amino acid residues (1950 aa) than other *Notch1* homologs (2531 aa, mouse; 2508 aa, coelacanth; 2437 aa, zebrafish) and 18 EGF-like repeats (typically 36 in other species) in the extracellular region. These characteristics are unlikely to be sufficient for ligand binding. In contrast, the *Notch2* homolog in elephant sharks seems functional, suggesting that only the Notch2-like receptor can be expressed as a functional receptor in thymic progenitor cells in elephant sharks. Therefore, in the thymus of elephant sharks, only the interaction between Dll1 and Notch2 is expected to transduce Notch signaling essential for T-cell development. In this study, we demonstrated that Dll1 triggers Notch signaling *via* Notch2 to induce the development of mature T cells, and it is the only kind of Dll-mediated Notch signaling present in elephant sharks, where the thymus primordium was first observed.

## Discussion

The thymus is thought to have emerged in jawed vertebrates—the common ancestors of cartilaginous fishes and bony vertebrates. As the *Dll4* gene, which encodes the essential Notch ligand for T-cell development in bony vertebrates, has never been found in the elephant shark (*C. milii*), likely, Dll1 emerges first as a Notch ligand in the thymus. In this study, we showed the potential of Dll1 to support T-cell development with both Notch1 and Notch2, whereas Dll4 preferentially cooperates with Notch1 in the murine thymus. In jawed vertebrates, before the coexistence of the two Notch receptors was established, Dll1 may have had advantages in triggering Notch signaling.

We confirmed that the *Dll4* gene in the coelacanth encodes a Notch ligand with distinctive features in two regions, DOS and MNNL, which are required for murine Dll1 and Dll4 to bind Notch, respectively. Therefore, like the murine Dll4-based chimera with the Dll1-derived DOS motif (21), the coelacanth Dll4 should act as a hyperactive Notch ligand, as the murine Dll molecules only have one or the other motif. On the other hand, most Dll4 molecules in bony fishes seem to lose the functional MNNL but retain the DOS motif, which resembles that of murine Dll1. This information raises the possibility that the hyperactive Dll4, which emerged in the common ancestor of lobe- and ray-finned fishes, weakened its activity during evolution to tetrapods and bony fishes *via* different mechanisms. Gain-of-function mutations of Notch receptors induce malignant transformation in various cell types (32); thus, limiting the intensity of Notch signaling to a certain range may be advantageous. Interestingly, the proximity between the appearance of the *Dll4* gene and the precise beginning of the coexistence of *Notch1* and *Notch2* seems to be related to the fact that Dll1 and Dll4 cooperate with Notch1/Notch2 and selectively with Notch1, respectively. Subsequently, the combination of Dll4 and Notch1 to induce stable Notch signaling is preferentially utilized, as seen in the induction of T-cell development (21).

Based on several findings regarding the significance of Notch receptor–ligand interactions in the development of various organs, it is clear that Dll4 binds to Notch1, whereas Dll1 does not distinguish between Notch1 and Notch2 and functions equally well with both (18). During vascular development, Dll1-mediated Notch1 activation is essential for the maintenance of arterial identity during late-stage arteriogenesis in mouse fetuses (13.5 days of gestation, E13.5) (33). Dll1 and Notch1 are also important for somitogenesis (34, 35). In both cases, Dll4 could not completely compensate for the loss of Dll1, suggesting a functional difference between Dll1 and Dll4 (36–38). In contrast, as both Dll1 and Notch1 are necessary for retinal development and Dll4 could substitute for Dll1 function, there was functional redundancy in the retina (37–39). In this study, the exogenous expression of Dll1 in the thymic epithelium in place of Dll4 supported T-cell development with similar cellularity, suggesting functional overlap between them. Thus, their functional differences were context dependent and might be due to the difference in the threshold amount of Notch signaling required. Reportedly, both Dll1 and Notch2 are essential for the appearance of marginal zone B (MZB) cells in the spleen, indicating that Dll1 cooperates with Notch2 to transduce Notch signaling (19, 25). In the spleen, Dll1 is expressed on stromal cells in the follicles and encounters the precursors of MZB cells or MZB cells that express Notch2 to activate Notch signaling that determines or maintains their cell fate (40). In contrast, Dll4 functions with Notch1 at an early stage (around E8.5) for the specification of arterial fate during vascular development in mice. In our study, neither endogenous nor exogenous Dll4 function with Notch2. Therefore, it was suggested that Dll1 can interact with both Notch1 and Notch2, in contrast to Dll4 that only acts as a functional ligand for Notch1 in the thymus. Significant contribution of the Notch2–Dll4 interaction has not been reported in lineage specifications of other organs, too. We have previously shown that interaction between Dll4 and Notch2 *in vitro* is clearly detected and other Notch–Dll combinations using Notch1- or Notch2-expressing cells stained with soluble form of the extracellular regions of Dll1 or Dll4 (21). Thus, dysfunction between Notch2 and Dll4 seems to be observed only *in vivo*, and there would be some unknown mechanisms underlying their inefficient interaction.

Using *in vitro* cultures with a monolayer of stromal cells, it was shown that Dll1 but not Dll4 induces Notch signaling *via* Notch2 that is sufficient for the specification of T-lineage cells (17). However, Dll1-mediated Notch2 signaling was not sufficient to drive T progenitors into the DP stage, and BM progenitor-derived T-lineage cells arrested their differentiation at the DN3 stage because of the impaired expression of the TCR β chain. In this study, the transition from DN to DP stage was completely restored, and mature SP T cells were also observed in iD1 mice with *Notch1*-deficient BM progenitors. These results suggested that Dll1-mediated Notch2 signaling can support the expression of the TCR β chain necessary for differentiation into DP and SP stages. This discrepancy may reflect the high capacity of the native thymic environment to support T-cell development. However, the efficiencies of the appearance of DP thymocytes derived from *Notch1*-deficient BM progenitors in iD1 mice were, in some cases, less than those derived from Notch1-bearing WT BM progenitors. In addition, stage-specific deletion of Notch receptors revealed that Notch1 is the main transducer of Notch signaling in DN2b/DN3 stages, while Notch2 has minor cooperative effects on Notch target genes (27). These differences can be attributed to the lower expression of Notch2 at the DN3 stage than that of Notch1 (16). Therefore, the downstream impact of the Dll1 and Notch2 interaction may not be identical to that of Dll4 and Notch1 in the thymus.

In conclusion, we showed here that Dll1 was supporting T-cell development with ancient Notch receptors when the thymus emerged and was replaced by Dll4 to trigger Notch signaling *via* Notch1 during their evolution. The latter combination might have some functional advantages in inducing T-cell development in the thymus.

## Materials and Methods

### Phylogenetic Analysis

We performed an evolutionary analysis using the maximum likelihood method. The evolutionary history was inferred using the maximum likelihood method and the JTT matrix-based model (41), and the tree with the highest log likelihood (−17,502.39 for *Dll*, −55,656.23 for *Notch*) is shown. The percentage of trees in which the associated taxa were clustered together is shown next to the branches. Initial trees for the heuristic search were obtained automatically by applying neighbor-join and BioNJ algorithms to a matrix of pairwise distances estimated using the JTT model and then selecting the topology with a superior log likelihood value. The tree was drawn to scale with branch lengths measured as the number of substitutions per site. This analysis involved 20 *Dll* and 18 *Notch* amino acid sequences. We obtained a total of 937 *Dll* and 2,848 *Notch* positions in the final dataset. Evolutionary analyses were conducted using MEGA11 software (42, 43).

### Mice

*Dll4^f/f^ Foxn1-cre* mice have been described previously (4). These mice were bred with iD1 or iD4 transgenic mice that retained the CAG promoter-driven *floxed GFP* and *Dll1* or *Dll4* cDNA at the *Rosa26* locus (21). *Notch1^f/f^ Rosa26^CreERT2^* or *Notch2^f/f^ Rosa26^CreERT2^* mice with the CD45.1 allele (16, 25, 30, 31, 44) were maintained in our laboratory. To delete the floxed genes, we administered tamoxifen (100 mg/kg) to the mice by i.p. injection four times on separate days. One week after treatment, BM cells were obtained and used as a source of *Notch1*- or *Notch2*-deficient hematopoietic cells for transplantation. GFP transgenic mice were originally established by our group (45). All mice were maintained under specific pathogen-free conditions, and the animal experiments were approved by the Animal Experimental Committee (Tokai University, Kanagawa, Japan).

### BM Transplantation

For BM reconstitution experiments with *Notch2*-deficient BM cells, semi-lethally irradiated (6 Gy) *Dll4^f/f^* (Cont), *Dll4^f/f^ FoxN1-Cre* (D4KO), iD4 *Dll4^f/f^ FoxN1-Cre* (iD4/D4KO), or iD1 *Dll4^f/f^ FoxN1-Cre* (iD1/D4KO) mice (CD45.2) were transplanted intravenously with BM cells from age-matched *Notch2^f/f^* (1 × 10^7^, CD45.1, represented as WT in the figure) or *Notch2^f/f^ Rosa26^CreERT2^* mice (1 × 10^7^, CD45.1, represented as Notch2 KO), who had been administered tamoxifen 1 week before the experiments and analyzed at 6 weeks after reconstitution. For those with *Notch1*-deficient BM cells, semi-lethally irradiated C57BL/6 mice were transplanted with BM cells from age-matched *Notch1^f/f^* (5 × 10^6^, CD45.1, represented as WT in the figure) or *Notch1^f/f^ Rosa26^CreERT2^* (5 × 10^6^, CD45.1, represented as Notch1 KO) mice, with GFP-transgenic (5 × 10^6^, CD45.2) mice as an internal control. After 4 weeks, the mice were administered tamoxifen, and 1 week after the treatment, BM cells were prepared for secondary transplantation into the recipient mice as described above.

### Flow Cytometric Analysis

For flow cytometric analysis, the following monoclonal antibodies (mAbs) and reagents were used: BV650-CD4 and PE-Cy7-CD3 (BD Biosciences, Tokyo, Japan); PE/Cy7-CD4, BV510-CD4, APC-CD8, APC/Cy7-CD8, Alexa700-CD8, FITC-CD11b, PerCp/Cy5.5-CD21, PE-CD23, PE-CD45.1, PE-CD45.2, FITC-B220, APC-B220, Pacific Blue-Thy1.2, Pacific Blue-c-Kit, APC-IL7Rα, PE-EpCAM, FITC-Gr-1, PE/Cy7-Sca-1, PE-Hamster IgG, PE-Notch1, PE-Notch2, PE-Notch3, and PE-Notch4 (BioLegend, Tokyo, Japan); and PerCp/Cy5.5-CD4, PerCp/Cy5.5-CD19, PE-Thy1.2, APC-Thy1.2, APC-PDGFRα, and FITC-TER119 (Thermo Fisher Scientific, Tokyo, Japan). PE/Cy7-CD19 and APC/Cy7-CD45.1 (Thermo Fisher Scientific). Stained cells were measured using FACSVerse (BD Biosciences) or FACSFortessa (BD Biosciences). Data were analyzed using FlowJo software (BD Biosciences).

### Immunohistochemistry

Immunohistochemical analysis was performed as previously described (4). Tissue sections of thymus were stained with FITC-anti-pan-cytokeratin (Sigma-Aldrich, Tokyo, Japan) and rabbit anti-HA Ab (Bio-Rad, CA, US) antibodies. Visualization was performed with Alexa594-anti-rabbit IgG antibody (Thermo Fisher Scientific). The stained slides were observed with LS880 (ZEISS).

### Statistical Analysis

Statistical analyses, as indicated in the figure legends, were performed using GraphPad Prism software (version 5.0; GraphPad Software, Inc., San Diego, CA). Differences between the two groups were evaluated using Student’s *t*-test for parametric samples and Mann–Whitney *U*-test for non-parametric samples. Statistical significance was set at p<0.05.

## Data Availability Statement

The original contributions presented in the study are included in the article/**Supplementary Material**. Further inquiries can be directed to the corresponding author.

## Ethics Statement

The animal study was reviewed and approved by the Animal Experimentation Committee of Tokai University.

## Author Contributions

KiH, HH, and KH designed and performed all the experiments, analyzed the data, and wrote the manuscript. YT, JI, MY, NN, and TS performed the experiments and supported data collection. MT performed the phylogenetic analysis. MO provided the GFP mice and supported the animal experiments. KA and SH supervised this study.

## Funding

This work was supported by grants from the JSPS KAKENHI [Grant number: 16K08848, 17H05802, and JP20K07330 (to KH)], the JSPS KAKENHI (Grant number: JP19H03692), The Uehara Memorial Foundation, The Naito Foundation, the Takeda Science Foundation, the Daiichi Sankyo Foundation of Life Science, the Tokyo Biochemical Research Foundation, the Research Grant of the Princess Takamatsu Cancer Research Fund, the Foundation for Promotion of Cancer Research, and The Mitsubishi Foundation (to HH), and the 2020 Tokai University School of Medicine Research Aid (to KiH, HH). This work was also partly supported by Research and Study Project of Tokai University General Research Organization to H.H.

## Conflict of Interest

The authors declare that the research was conducted in the absence of any commercial or financial relationships that could be construed as a potential conflict of interest.

## Publisher’s Note

All claims expressed in this article are solely those of the authors and do not necessarily represent those of their affiliated organizations, or those of the publisher, the editors and the reviewers. Any product that may be evaluated in this article, or claim that may be made by its manufacturer, is not guaranteed or endorsed by the publisher.

## Supplementary Material

Supplementary Figure 1 Phylogenetic analysis of *delta-like (Dll) 1* and *Dll4* genes. Homologs of *Dll1* and *Dll4* genes are sorted from the NCBI database in terms of their homology to murine *Dll1* and *Dll4* genes. The evolutionary history was inferred using the maximum likelihood method and the JTT matrix-based model (41). The tree with the highest log likelihood (-17502.39) is shown. The percentage of trees in which the associated taxa were clustered together is shown next to the branches. The tree was drawn to scale with branch lengths measured as the number of substitutions per site. The analysis involved 20 amino acid sequences. There were 937 positions in the final dataset.

Supplementary Figure 2 The numbers of thymocyte subsets. Thymic cellularity (mean ± SD) of thymocyte subsets (CD4^+^CD8^+^, DP; CD4^+^CD8^-^, 4SP; CD4^-^CD8^+^, 8SP; CD4^-^CD8^-^, DN; CD19^+^, CD19) derived from the mice in **Figure 2** were shown. **P<0.01, *P<0.05 by Student *t*-test.

Supplementary Figure 3 Bone marrow (BM) chimera experiments. The experimental schemes for BM chimeras are shown for **Figures 4A** and **5B**.

Supplementary Figure 4 Mature T cells from Notch1-deficient(Italic) BM cells in the lymph nodes of iD1 and iD4 mice. All data were obtained from inguinal lymph nodes of the BM chimera, as shown in **Figure 5**. **(A)** Flow cytometric analysis of inguinal lymph nodes from secondary BM chimeric mice. Numbers in the profiles indicate the relative percentage of CD45.1^+^ or GFP^+^ cells (internal control, Int. cont) (left panels, B220 vs. CD3) and CD45.1^+^CD3^+^ or GFP^+^CD3^+^ cells (right panels, CD4 vs. CD8) for each quadrant or fraction. The results represent at least three independent biological replicates. **(B)** The efficiency of CD3^+^ T cells derived from control (WT) or *Notch1*-deficient (N1KO) BM cells was examined. The T cell appearance index was calculated as the ratio of CD45.1^+^/GFP^+^ CD3^+^ T cells and CD45.1^+^/GFP^+^ B220^+^ B cells in the inguinal lymph nodes (mean ± SD; WT as donor; Cont, n=3; D4KO, n=3; iD4/D4KO, n=3; iD1/D4KO, n=4; N1KO as donor; Cont, n=5; D4KO, n=5; iD4/D4KO, n=5; iD1/D4KO, n=5). Data were collected from three independent experiments. Each closed circle indicates the index for each mouse. **P<0.01, ***P<0.001 using Mann–Whitney *U* test.

Supplementary Figure 5 Phylogenetic analysis of *Notch1* and *Notch2* was performed as shown in **Supplementary Figure 1**. The tree with the highest log likelihood (-55656.23) is shown. This analysis involved 18 amino acid sequences with a total of 2848 positions in the final dataset.
