# Supplementary figures and images for: Dll1 Can Function as a Ligand of Notch1 and Notch2 in the Thymic Epithelium
Source: Front Immunol. 2022 Mar 17;13:852427. doi: 10.3389/fimmu.2022.852427 (PMC8968733; doi:10.3389/fimmu.2022.852427)

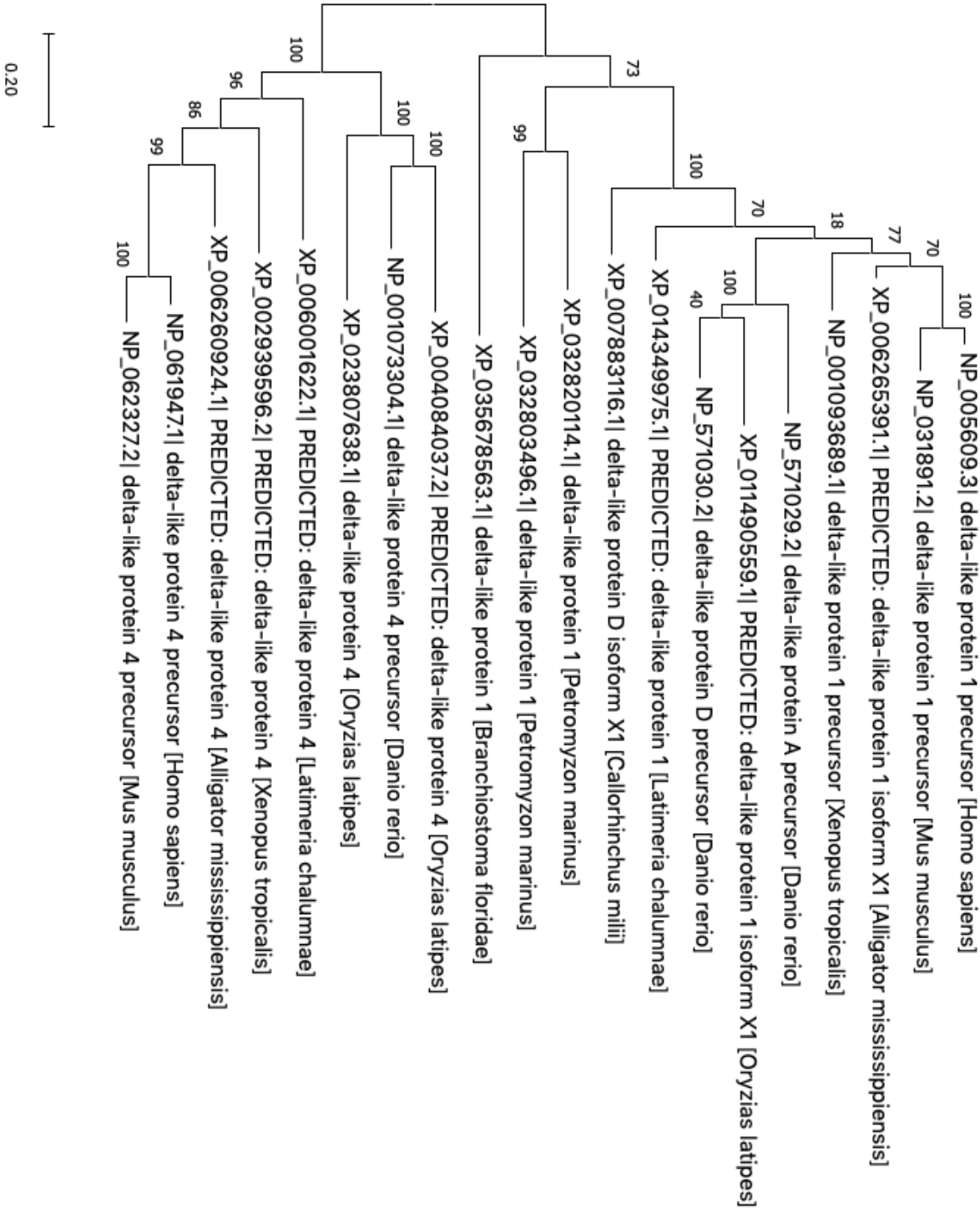

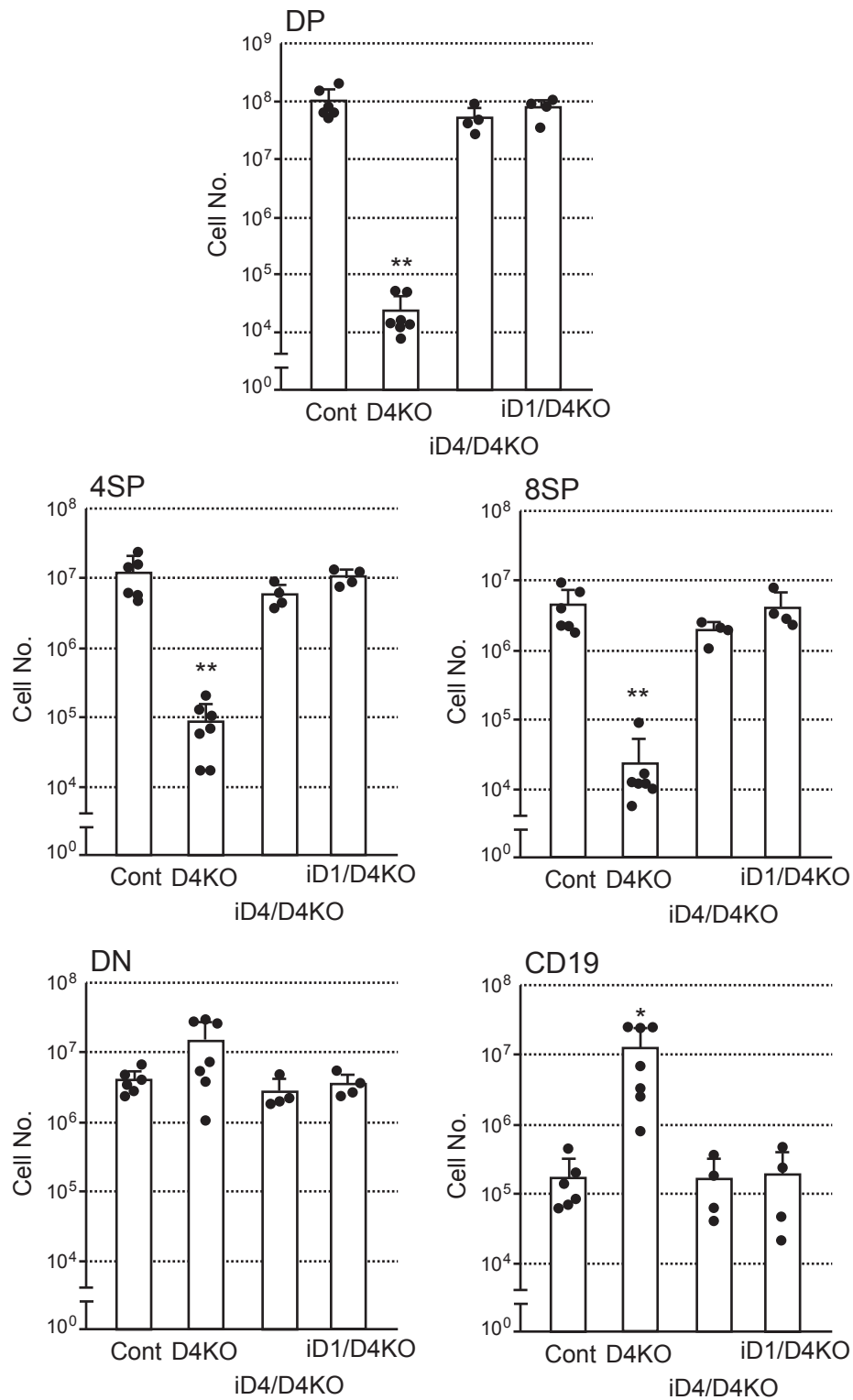

**A**

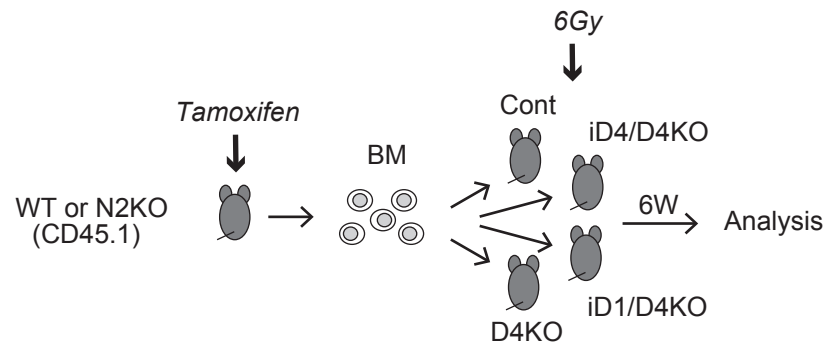

**B**

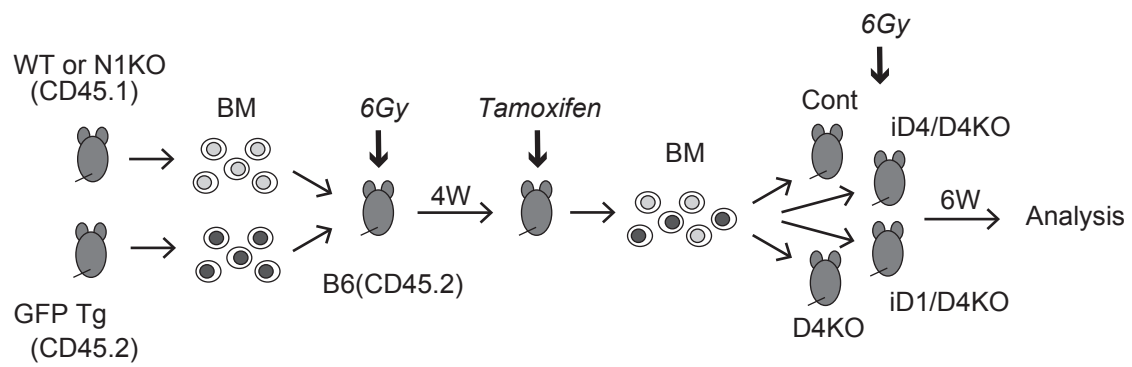

**A**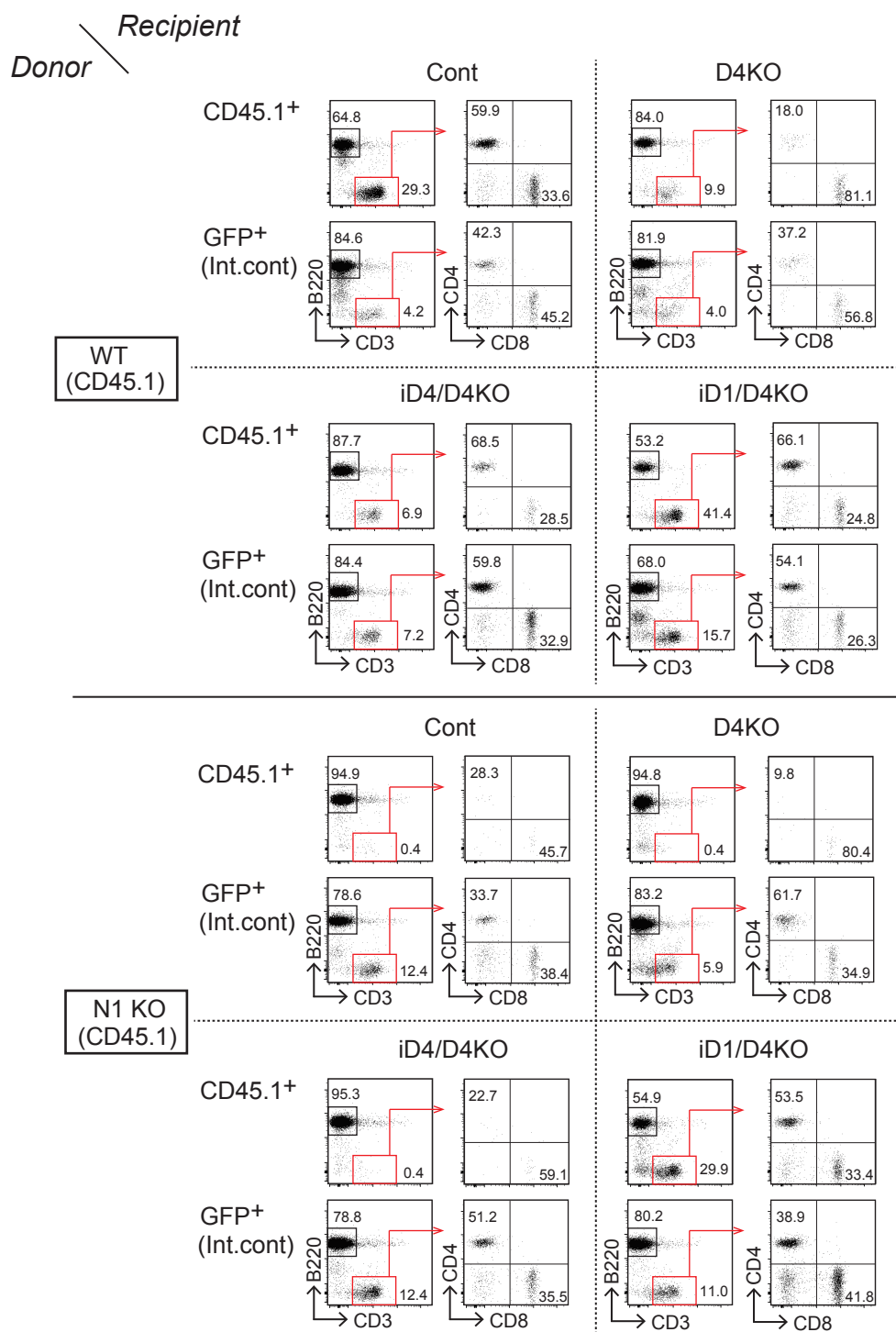

**B**

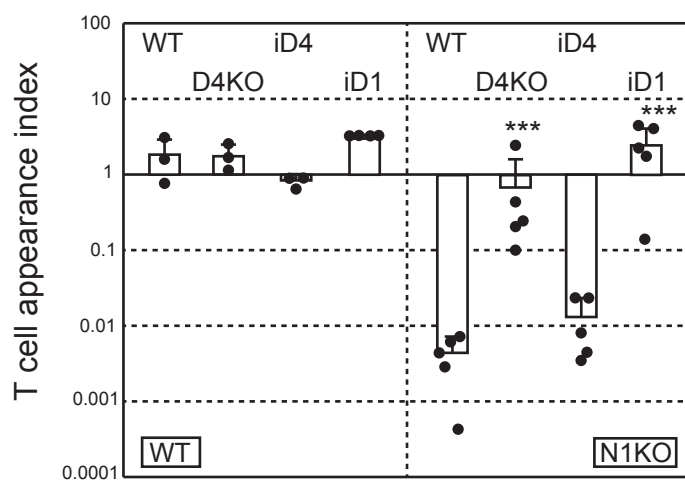

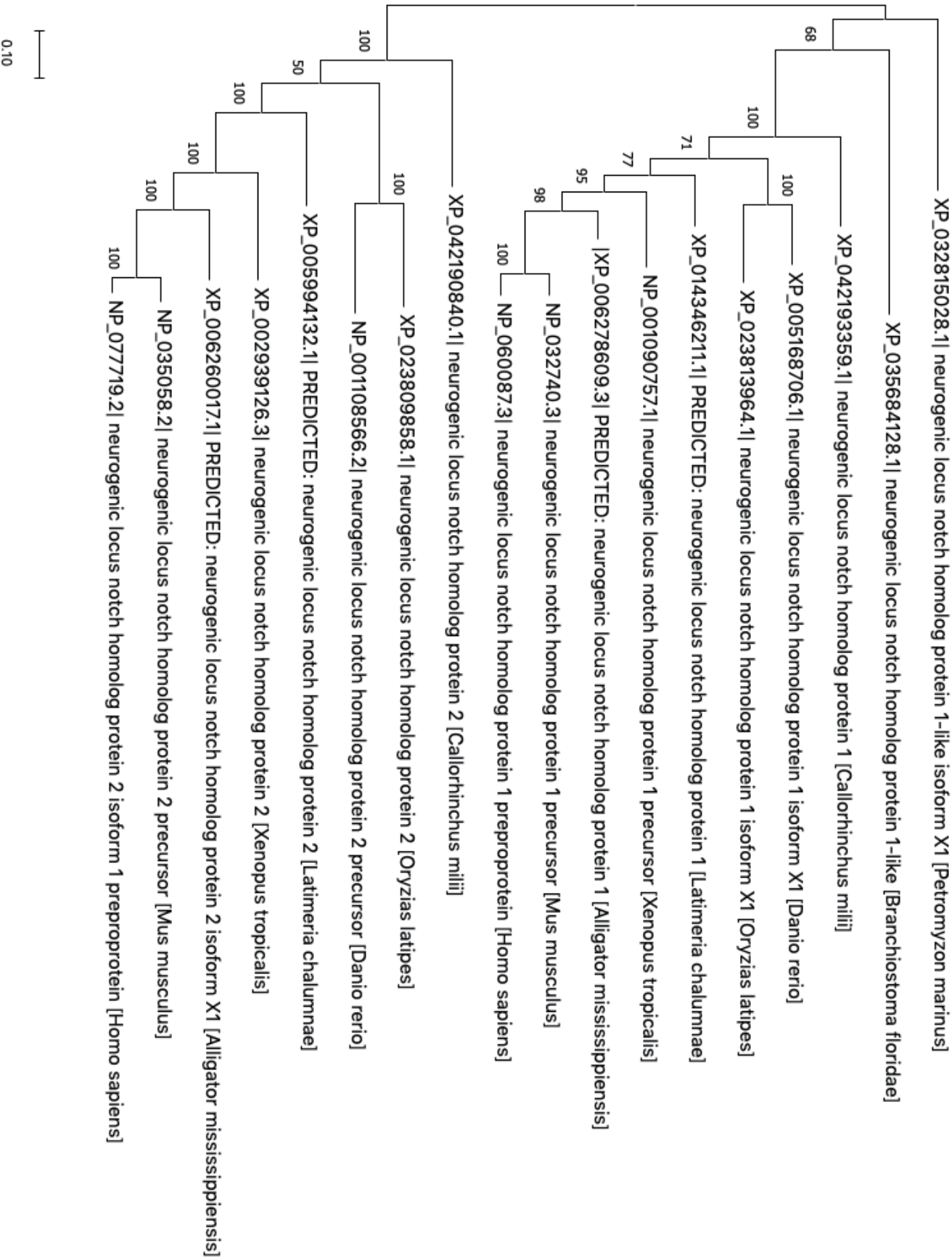

Supplement: Supplementary Figure 1 — Phylogenetic analysis of delta-like (Dll) 1 and Dll4 genes. Homologs of Dll1 and Dll4 genes are sorted from the NCBI database in terms of their homology to murine Dll1 and Dll4 genes. The evolutionary history was inferred using the maximum likelihood method and the JTT matrix-based model (41). The tree with the highest log likelihood (-17502.39) is shown. The percentage of trees in which the associated taxa were clustered together is shown next to the branches. The tree was drawn to scale with branch lengths measured as the number of substitutions per site. The analysis involved 20 amino acid sequences. There were 937 positions in the final dataset. [file Image_1.pdf]
